# Development of a solar powered multirotor micro aerial vehicle

**DOI:** 10.1038/s41598-024-54079-9

**Published:** 2024-03-08

**Authors:** Aly Abidali, Stephen A. Agha, Antonio Munjiza, Mohammad H. Shaheed

**Affiliations:** 1 School of Engineering and Materials Science, Queen Mary University of London, London, E1 4NS UK; 2 Department of Engineering and Maths, Sheffield Hallam University, Sheffield, S1 1WB UK

**Keywords:** Energy science and technology, Engineering

## Abstract

Rotary-wing aerial vehicles offer manoeuvrability and vertical take-off and landing (VTOL) advantages over fixed-wing systems. Rotary-wing systems do however have comparatively higher energy demands and consequently shorter flight times and therefore a greater energy dependence over their fixed-wing counterparts. Advances in photovoltaic technologies have resulted in significant increases in the specific power (power-to-weight-ratio) of solar cells enabling the design of solar-powered rotary-wing aircraft, and now micro-sized variants. The micro aerial vehicle (MAV) presented, the Micro Solarcopter, is a 0.15 m $\times$ 0.15 m $\times$ 0.02 m solar-rechargeable radio-controlled aircraft. The 0.071 kg aircraft can fly for an average time of 3.5 min, recharge in approximately 68 min under 1000 W/m^2^ irradiance at 25 °C and can hibernate for 38 days without sunlight. The paper explores the use of commercially available photovoltaic cells for the purpose of increasing the energy autonomy of multi-rotor MAVs, by enabling them to stay out in the field without returning to base for charging. A working prototype has been presented which incorporates a battery management system, automatic power on and off, low-power sleep mode, and first-person-view (FPV) camera.

## Introduction

Project sunrise was the first solar-powered fixed-wing aircraft to take to the skies^1^. It was designed over 40 years ago by Roland Boucher in 1974. Since its inception, many more designs of successful fixed-wing solar-powered aircraft have been made, such as the Sky-Sailor^2^, Zephyr^3^, AtlantikSolar^4^, Solar-Impulse, and Facebook’s Aquila drone in 2016^5^. All of these utilise fixed-wings, and most are designed for high altitude long endurance missions.

The first solar-powered multi-rotor began development in 2011 and first flew in 2012, utilising a charge controller, solar panel, and battery^6^. Following this multi-rotor came the development of the Solarcopter which was the first solar-powered multi-rotor aircraft to fly utilising only the sun’s energy, without any means of energy storage; the sun shines, and the vehicle flies by converting sunlight directly to propulsion without any batteries on-board^7^. The Solarcopter project demonstrated the potential of solar power in multi-rotors by proving that a quadrotor design can fly utilising the sun’s power directly. Other examples of solar-powered multi-rotors include those developed by Lachica et al*.*^8^, Pramod^9^, and by students at the National University of Singapore^10,11^. The Mars Helicopter developed at the National Aeronautics and Space Administration (NASA) is the first solar-powered rotorcraft to demonstrate the viability and potential of heavier-than-air vehicles in the Martian atmosphere and was launched in 2021^12^. Elkunchwar et al.^13^ have also demonstrated a solar-rechargeable crazy-fly quadrotor with two 0.093 m $\times$ 0.073 m panels that fold during flight.

**Figure 1:**
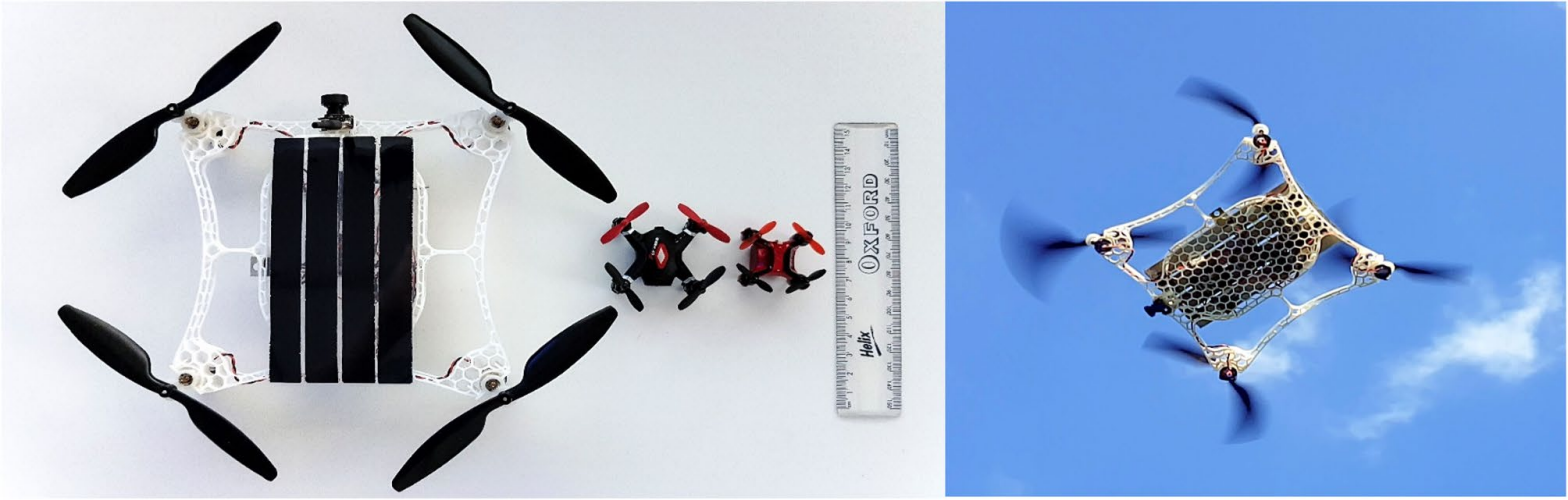
Micro Solarcopter size comparison (left) and the aircraft in flight (right). With a weight of 0.071 kg and a footprint of 0.15 m $\times$ 0.15 m without rotors, the Micro Solarcopter is presently the smallest flying solar-powered multi-rotor MAV. Beside the Micro Solarcopter in the left image are two of the smallest commercially available quadrotors; the M:Tech Micro and the Skyline Nano with footprints of 0.04 m $\times$ 0.04 m and 0.03 m $\times$ 0.03 m respectively.

These solar-powered rotorcraft are relatively small when compared to conventional solar fixed-wing aircraft but large when compared to the most miniature aerial robots currently developed such as the Mesicopter^14^ and Black Widow MAV (Micro Aerial Vehicle), where a MAV has generally been defined as having a span of less than 6 inches (0.1524 m), and a mass of less than 0.1 kg^15^. Larger rotorcrafts are unsuitable for certain applications that require close-quarter or low-level flying such as maneuvering around tight spaces inside buildings or in the natural environment through caves or dense areas of vegetation. Size also limits the swarm flying capabilities of these large aircraft due to their inherent lower agility as a result of their greater mass moment of inertia and their higher cost compared to smaller systems^16^. The Micro Solarcopter (Fig. 1) is the smallest solar-rechargeable multi-rotor developed to combat some of these limitations.

Conventional applications include building inspection, wildlife tracking, weather monitoring, or to operate as atmospheric satellites. Solar multi-rotor MAVs also show potential for use in areas where access to power is rare or non-existent, such as in offshore missions, deserts, or exploration of other planets such as Mars, depending on the atmospheric and gravimetric properties of the planet^19^. Solar power is an abundant renewable energy source capable of providing significant environmental benefits in comparison to conventional energy sources^20^. Incorporating solar power will enable energy autonomy for these MAV systems and utilising them in swarms rather than a single larger system has the potential to improve a mission’s success rate as it will not be dependent on a single aircraft.

## Conceptual design considerations

A quadrotor platform was chosen as it is mechanically simple^22^ and provides an ideal central area for placement of a solar panel^7^.

The single cell specification of the SunPower cell used in the micro Solarcopter prototype are summarised in Table 1. The specific power for this cell is $543\mathrm{ W}/{\text{kg}}$, with a power per unit surface area of $219\mathrm{ W}/{{\text{m}}}^{2}$ under the 1000 W/m^2^ solar irradiance condition. The mass per unit surface area for the SunPower solar cell is $0.40\mathrm{ kg}/{{\text{m}}}^{2}$. Theoretically, the Shockley Queisser limit predicts an 86% maximum solar conversion efficiency^24^. Consider a solar cell operating at this theoretical power conversion efficiency and the surface area of the M:Tech quadcopter (see Fig. 1) of 0.04 m × 0.04 m; the maximum solar cell power output will be 1.38 W at 1000 W/m^2^ solar irradiance.

**Table 1:** Single cell properties for a SunPower C60 cell^23^.

Parameter	Value
Peak power at 1000W/m^2^ and 25 °C	3.42 W
Current at maximum power point	5.93 A
Voltage at maximum power point	0.582 V
Single cell efficiency	22.5%
Surface area	0.0156 m^2^
Mass (unencapsulated)	0.0063 kg
Cell thickness	0.000165 m

The M:Tech quadcopter is one of the smallest commercially available systems in the MAV class and is analysed here as an indication of the applicability of solar-powered flight of a rotary-winged aerial vehicle at this scale. Its electrical power consumption at hover was measured to be approximately 3.57 W. Therefore, the minimum power requirements of the M:Tech drone at hover is 2.59 times the output provided by this theoretical solar cell meeting the surface area size requirements of the MAV. The limiting factors here are the solar irradiance and the solar power conversion efficiency.

The solar panel mass cannot exceed $0.0035 {\text{kg}}$ if it is to replace the battery mass of the M: Tech. Therefore the specific power of the solar cell assuming no power losses for the hover condition of the M:Tech quadcopter will be $1020\mathrm{ W}/{\text{kg}}$. Now assuming that the specific power requirement could be met by the SunPower cell (which in reality it cannot), based on this solar cell’s power per unit surface area of $219\mathrm{ W}/{{\text{m}}}^{2}$ and the M:Techs hover power of $3.57 {\text{W}}$, the minimum solar panel surface area required for purely solar-powered flight would be 0.128 m × 0.128 m. For the Micro Solarcopter to hover purely on solar power, it will need about 12W, therefore requiring a surface area of 0.234 m × 0.234 m, which is outside the MAV size restriction.

The system mass has also been assumed to remain constant while in reality the mass of the frame will increase with size. Furthermore, if this larger solar panel were to replace the combined mass of the battery and solar panel currently on the system (13.5 g) then this would require the solar panel to have a specific power of 889 W/kg. While solar cells with this specific power exist, they do not necessarily have adequate power density as a panel (i.e. power per unit area), and cannot necessarily be sized in this way as there are clearance requirements between connected cells to consider when forming a panel.

These limitations make a purely solar-powered multirotor MAV at the scale of the M:Tech quadcopter unfeasible, and at the scale of the Micro Solarcopter highly impractical with current technology. The conclusion is therefore that at the MAV scale, the payload and usable surface area of quadcopters are too small to achieve solar-powered flight without energy storage mainly due to performance limitations of current solar cells and propulsion technology.

Furthermore, total reliance on solar power alone would render the system susceptible to the inexorable variations in solar power over time in the outdoor environment. This would make the aircraft unstable in flight due to power fluctuations. In addition, a solar panel captures maximum power when its light-absorbing surface is orientated perpendicular to the sun^25^.

The effective irradiance varies with the cosine of the incidence angle between the solar panel and the sun. Hence as the aircraft maneuvers, its orientation in space affects the power generated, due to this change in incidence angle for the panel which is fixed to the aircraft frame^26^. Finally, any shadows on the panel’s surface caused by clouds, or other objects in the environment will also affect the panels output power, and if this is lower than the propulsion systems power requirements, then the aircraft would not be able to sustain flight.

**Figure 2:**
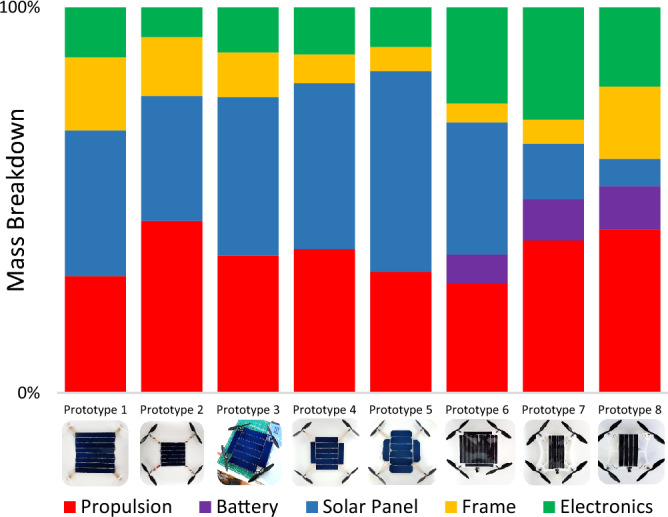
Mass breakdown of critical prototype components. A total of eight prototypes were made before a working model was reached. The first five prototypes were attempts to produce a solely solar-powered quadcopter without batteries. The only flying prototype was prototype 8 which is presented in this article.

Prototypes 1–5 presented in (Fig. 2) are the attempts at developing a purely solar-powered MAV without energy storage. Development of Prototype 1 was focussed on minimising system mass, including directly coupling the solar panel to the flight controller (no charge controller). The solar panel mass was however underestimated and the power requirement for flight was not met. Prototype 2 utilised a more efficient, but heavier propulsion system with a resized solar panel to match its power demands. Power requirement was still not met by the solar panel resulting in voltage sag. Prototype 3 had double the solar panel output of prototype 2 but with only a 23.3% increase in mass due to a light but fragile frame. Thrust output was however reduced due to obstruction of propeller airflow as arm lengths were reduced. Prototype 4 had the same panel as the previous prototype but with a larger balsa wood frame resulting in 4.5% overall mass reduction but with a surface area of 0.25 m × 0.25 m. The solar power generated was still insufficient for flight. With the same frame as the previous iteration, prototype 5 had an even larger solar panel producing 32.9% more electrical power with 20.2% more overall mass. The infeasibility of purely solar-powered rotary-winged flight at this scale was reached at this stage.

Prototypes 6–8 were developed with energy storage. The micro Solarcopter presented in this article (prototype 8) can therefore be referred to as a solar-rechargeable MAV. Prototype 6 had a laser cut polystyrene frame and larger solar panel relative to prototype 7 and could not escape ground hover. The panel for prototype 7 was reduced and the frame was further reduced in mass and Arduino Beetle control electronics for hibernation was changed to the ATtiny85. This prototype also did not fly and the polystyrene frame was not sufficiently robust due to excessive vibration. Prototype 8 was therefore redesigned and strengthened using 3D printed PLA. The solar panel was also sized based on specific power via improved tracking efficiency. These changes were necessary and sufficient for prototype 8 to fly beyond ground effect.

The sizing of the propulsion system is primarily dependent on the desired take-off mass and size restriction of the entire system^27^. Ideally all propeller parameters would be refined for this specific application. The propeller would then be manufactured from a light weight, rigid material, such as carbon fibre, and then coupled with a motor and possibly a gearbox. This will be to ensure that the required propeller torque and rotational speed for maximum propeller thrust efficiency is reached at the maximum motor efficiency. In this study, coreless motor technology was selected for its light weight and rapid response to flight control signals, ensuring stable flight can be achieved by this fly-by-wire aircraft. These motors were coupled with existing commercially available micro drives and propellers that met the MAV size restrictions and were assessed on thrust output vs power input. The weight of each propulsion system was subtracted from the thrust generated to ensure that this was factored into the assessment. This was then followed by selecting a suitable energy storage solution, which was a single cell lithium polymer chemistry battery for its high energy density. The electronics for power regulation and flight control were then determined followed by an appropriately sized solar panel as described in the methods section. Finally, a suitable structure to house all the components of the system was designed. Please see supplementary S1 for more details regarding components.

## Methods

### Propulsion

The primary objective of this study was to produce a solar-powered multi-rotor MAV. To achieve this an estimated solar-panel electrical power output was determined based on present cell technologies for a solar-panel that would fit within MAV size restrictions. Several propeller, motor, and gearbox combinations were then tested for maximum thrust output based on this power input. The effects of scaling down the system were also considered when designing these propulsion systems. The scaling down of electromagnetic motors, for instance, tends to come with an overall reduction in efficiency^14,19^. In addition to this the lift to drag ratio tends to reduce drastically for Reynolds numbers lower than 10^5^^28^, so scaling down propellers is also not advantageous. It is therefore crucial that the propulsion system is accurately sized with respect to the size and mass constraints of the entire system. At low Reynolds numbers, there are higher mechanical power losses due to friction, as viscous forces dominate inertial forces resulting in a relatively greater propeller drag moment compared to larger systems. Increasing the propeller's diameter within the size restrictions of a MAV and lowering its pitch would, therefore, provide greater aerodynamic efficiency as the Reynolds number would increase and the lower pitch aids in keeping the boundary layer around the propeller attached to the aerofoil.

A low disk loading would achieve a low power loading (ratio of required rotor mechanical power to rotor thrust)^29^, or high power loading (if defined as rotor thrust to rotor mechanical power ratio)^30^ further increasing aerodynamic efficiency.

Propulsion experiments were conducted on a 3D-printed rig scaled down for miniature motors. The rig consists of a 0–0.1 kg measuring range load cell, and HX711 load cell amplifier for measuring the thrust force, a voltage sensor for the motor voltage, and a current sensor for the motor’s current draw. The load cell was calibrated using a 0.02 kg precise mass. The data acquisition unit was produced using an Arduino Uno to bring all the components together. Plots of thrust output vs electrical power input were made for each propulsion system. The weight of the propulsion system was subtracted from the result to determine the useful thrust output for a fair comparison between systems, this was to ensure if one system had the same thrust output for the same electrical power input as another system, then the lighter of the two systems would be chosen. The average thrust per electrical watt was then calculated to act as an efficiency indicator as the final prototype weight and operating range was initially unknown. The average thrust per watt, with propulsion system weight factored in as explained, along with the maximum thrust generated and size of the system were the key takeaways used to determine suitability. The maximum thrust is important, as a propulsion system could be found to be more efficient relative to another, but unable to generate sufficient thrust for a prototype to fly. Please see supplementary S1 for additional details.

### Energy storage

If the aircraft is to sustain stable flight, it will have to maintain a stable voltage within a specified range for the electronics to operate correctly and must have sufficient current output to satisfy the power demands of the system, especially for propulsion, with limited voltage drop. A voltage drop/sag occurs when the power source is overloaded, and either cannot meet the power requirements of the load or is met with high resistance^31^ resulting in losses via Ohmic heating. The voltage of the system should ideally slowly drop until a cut-off voltage is reached at which point the battery is completely depleted. The resistance between the components can be reduced as much as possible to ensure that the energy source delivers the maximum useful power to the load efficiently^31^. There are four main criteria when considering an energy storage solution for a solar-powered multi-rotor MAV, specific power (the maximum power output of the battery per unit mass), the specific energy (stored electrical energy in watt-hours per unit mass), power density (maximum power output of the battery per unit volume) and energy density (stored electrical energy per unit volume). Lithium polymer batteries were determined to be the best storage solution based on the four criteria. Super capacitors were considered for the energy system given their ability to quickly absorb and release energy, but ruled out due to their low specific energy and energy density relative to lithium batteries. Further selection criteria may also include cycle life, robustness, thermal performance with regards to efficiency and operating temperatures.

The main performance measures for the battery candidates were capacity; determined by the current output per hour, operating voltage; determined by series cell count, continuous power output; revealing the maximum current the battery can continuously provide, the max power output; indicating the current the battery can provide for short durations and the max power input; revealing the maximum current the battery can be charged at and hence the fastest charging time possible. The battery volumes and masses were measured and based on the Amp hour capacity, and maximum discharge ratings of the batteries, the specific power, specific energy, power density and energy density values for each battery were determined. The discharge testing can then be conducted as shown in (Fig. 4) for any selected battery.

Ideally, the system should have a thrust to weight ratio of 2:1 for good flight stability and control. Systems are usually designed for a thrust to weight ratio of 2.2:1 with the extra 0.2 to make up for efficiency losses and to ensure that the hover point is at 50% throttle. Theoretically, however, the system should be able to fly with a thrust to weight ratio of more than 1:1. The mass of a prototype based on a selected propulsion system and battery could be estimated to determine the operating current of the propulsion system for the aircraft to fly based on this mass, and then the battery suitability could be assessed based on the total current draw and a flight time estimate can then also be made.

There is nothing that can be done to modify the battery's internal resistance, however, the wiring can be designed to minimise resistive losses. If the resistivity or length of wire used is halved, then the resistance is also halved. Selecting a suitable wire material such as copper or silver with an inherently low resistivity value can therefore reduce the power loss. The limit on the minimum length of wire needed is a function of the frame size, as the majority of wire is used to transmit power to the propulsion system motors. If the cross-sectional area of the wire is doubled, the resistance of the wire is halved, however the mass of the wire increases proportionally. Removal of the insulation can also increase performance by reducing mass and increasing heat dissipation at the cost of exposing the wire.

Ohmic loss across a length of wire is proportional to the square of the current flowing through it. For the same resistance, by halving the current, the power loss is reduced by three quarters. For the same power transmitted, the current can be reduced by increasing the voltage across the terminals, however, the voltage was limited by the requirements of the electronic and electromagnetic components which operate at low voltages. The system current draw, whose main contributor is the propulsion system, dictates the minum diameter of wire used. The maximum size is limited by the mass and maximum allowable voltage drop across the wire, which is effected by its length, diameter and resistivity due to the conductor material.

### Electronics

As quadrotors are fly-by-wire systems, a flight controller is required for stable flight^32^. These FCs continually alter the speed of each rotor to maintain a stable hover and directional control of the aircraft. FCs use feedback from an IMU (accelerometer + gyroscope) for linear and angular position/rates and possibly a magnetometer for heading with respect to the earth’s magnetic field. The values generated by these sensors are then processed through a microcontroller on the FC board which then corrects the attitude of the aircraft for stability and control. This is achieved by changing the rotational speed of the motors using electronic speed controller’s (ESC), of which one is assigned to each motor. These ESCs comprise solid-state relays also known as metal oxide semiconductor field-effect transistors (MOSFETs)^33^.

FC selection was based on searching for the smallest and lightest available controller for brushed type motors, which was compatible with a receiver that the transmitter available at the time could communicate with. The final selected FC was the HappyModel Sp racing F3 Evo brushed micro.

The F3 Evo was selected as it comprises a 32-bit microcontroller giving it greater processing power, and for its support of BetaFlight, a popular open source multi-rotor firmware. This flight controller also required a separate receiver to be installed, the Spektrum satellite receiver was selected for this purpose.

A Rigol® DS1054Z oscilloscope coupled with a Hantek® CC-65 AC/DC current clamp, UNI-T UT61E multimeter, and Voltcraft® PPS-13610DC power supply was used for debugging circuits. The scale used to measure masses of small components was the ON BALANCE CT-250, as it provides accuracy up to three significant figures for masses under 0.05 kg. A Sparkfun Tiny AVR programmer was used for programming the ATtiny85 microchip.

### Energy generation

The Micro Solarcopter achieves energy autonomy using solar energy, providing a great way of generating power without polluting the environment, after the carbon cost from manufacturing. There are various types of solar cells, but the three most common are; monocrystalline, polycrystalline, and thin-film amorphous^4^. The highest performing solar cells are multi-junction cells, but these are generally very expensive and, in some cases, not commercially available. The electric current output of a solar cell is a function of the irradiance of the sun and the temperature of the cell^34^. The SunPower C60's single-cell maximum power point output voltage (0.582 V) falls short of the 4.2–3 V range needed for the MAV's electronics and electro-mechanical components. Connecting cells in series to achieve the required voltage poses challenges due to the cells' natural size, surpassing MAV size restrictions. Experimentation demonstrated that segmenting cells into equal parts allows voltage buildup in series connections, albeit at the cost of reduced current output per cell segment. This approach enables the creation of a solar panel with optimal voltage and current output, considering factors such as mass, surface area, and propulsion power requirements. The width of a segment is influenced by propulsion system current demands, while the panel voltage, determined by the number of segments connected in series, can be tailored to meet both electronic hardware and propulsion system requirements, while maintaining a compact form factor. Solar panels were simulated on hardware using a Chroma 62000H-S series solar array simulator to ensure the consistency of solar array output during experimentation. The load on the panels was simulated using an Applent AT8612 DC electronic load. SunPower C60 cell parameters were input into the array simulator to match the IV-curve characteristics of this solar cell. The charge time test involved using the physical battery and charging chipset with the Chroma solar array simulator to simulate the tested solar panel under standard test conditions.

### Frame and solar panel fabrication

3D printed prototype frames were printed on a modified Wanhao D6 3D printer. The printer nozzle was converted to an all-metal design to print at higher temperatures required by specific 3D-printing materials such as carbon infused filaments. A 40W CO_2_ laser cutter was used for shaping some of the foam and wood frames for early prototypes. A solar cell cutting rig was manufactured using wood, a Dremel 3000 rotary tool coupled with a diamond cutting disc, and a Dremel 3-in-1 workstation. A thick metal ruler was used to guide the cell through the Dremel tool’s cutting disc. Note that the cutting process generates fine toxic particles, hence use of personal protective equipment is advised.

## The key components

**Figure 3:**
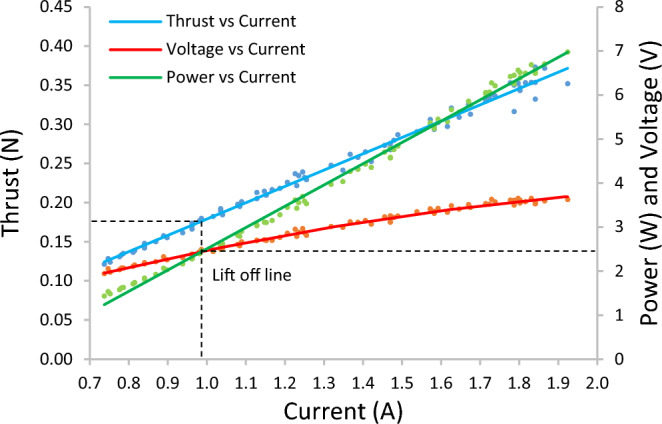
Selected Propulsion system properties for a single motor-propeller pair.

Through experimentation, a propulsion system was selected that utilized a Nine Eagles P-51 micro electric motor and gearbox coupled with a Turnigy Micro-quad propeller. The top end of the thrust band of this setup reached just over $363.0\times {10}^{-3}{\text{N}}$ ($\pm 0.5\times {10}^{-3}{\text{N}}$) of thrust. The static thrust test (Fig. 3) shows the hover power, current, and voltage of the Micro Solarcopter based on the mass of the prototype and the thrust output of the selected propulsion. The propulsion component is one of the most critical parts of the entire system as it greatly impacts the power required to achieve flight. As the Micro Solarcopter has a mass of 71.51 × 10^–3^ kg, its weight will be about 0.702 N. At the lift off-line, 1/4 of the weight is generated by each motor-propeller providing 0.175 N of thrust each. Therefore, the Solarcopter requires under 12W of electrical power at hover (excluding power requirements of all other electrical components).

**Figure 4:**
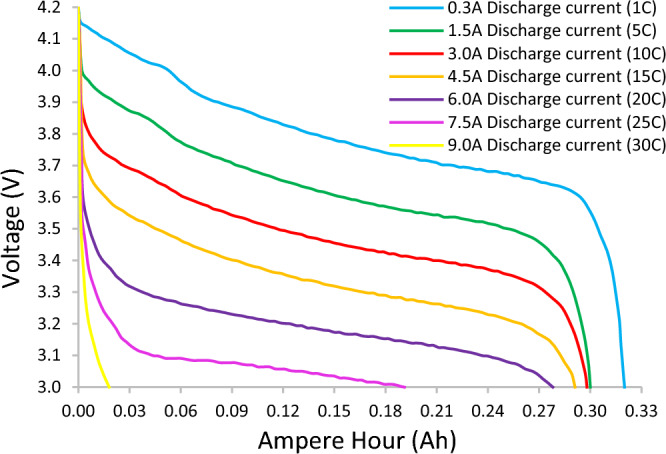
Selected battery discharge performance (Turnigy Nano-Tech 0.3Ah, 45–90C). For a given Ampere Hour value, a 3% maximum uncertainty in voltage between repeats at the same load setting was observed.

The performance of the propulsion system will dictate the size of the energy storage and solar panel components. The Micro Solarcopter was found to operate in a Reynolds number region between hover, and max power, defined as $\mathrm{14,500} < Re < \mathrm{19,600}$. For the hover condition, the disk loading of the Micro Solarcopter was calculated to be 13.84 N/m^2^ based on a 0.175 N thrust per propeller (Fig. 3) with radius 0.0635 m. The power loading was also estimated to be 0.109 N/W assuming a conservative 70% coreless motor efficiency and 95% spur gearbox efficiency giving a 1.61 W mechanical output power per propeller for a 2.42 W electrical input power to each motor at hover. The ratio of the battery's housing mass to the mass of the power generating part of the battery increases when scaling down in size. This results in an overall lower specific energy for smaller batteries compared to larger batteries of the same type. The Turnigy 0.3 Ah, 45–90 C battery was selected for the final design as it was the best match to meet the propulsion power requirements (discharge rate and capacity) while being compact and not increasing the mass of the entire system substantially. In terms of the key performance indicators, this battery had the best power density, and was third best for energy density compared to other candidate batteries. It was second best for specific power and fifth for specific energy. (see Fig. S1 in supplementary). The battery's performance at different loads (Fig. 4) shows that it did not reach its maximum advertised discharge C-rating. The battery is advertised as 45–90 C discharge capable, however, its performance drastically deteriorates beyond a 25 C constant current load. The Micro Solarcopter hovers at about 15 C load on this battery.

**Figure 5:**
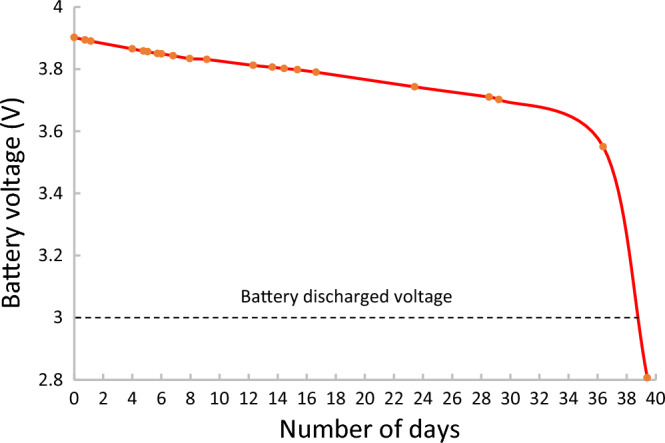
Battery discharge curve for vehicle in hibernation over 40 days with solar panel not exposed to light. The voltage drop of the battery from 3.9 to 2.8 V can be seen as it was discharged while the Micro Solarcopter was in sleep mode over a 40-day time period. The solar panel was disconnected from the system to prevent charging. As the safe voltage of the depleted battery is 3 V, the sleep time is estimated to be approximately 38 days.

The Micro Solarcopter enters a hibernation mode when its battery is depleted. This mode ensures that the aircraft does not drain the battery during periods of low or no light, such as during the night. While in this hibernation mode, a MOSFET is used to cut power going to the FC, propulsion and FPV camera, by means of a separate microcontroller. This microcontroller shuts down all non-critical modules within it, reducing power consumption further. The microcontroller wakes itself up every 8 s to check the battery voltage. If the voltage of the battery indicates full charge then the microcontroller turns all systems back on; if not, it will go back to sleep, and the process repeats. In this way, the Micro Solarcopter can sleep for long periods. (Fig. 5) shows the battery voltage variation over approximately 40 days when the vehicle is hibernating.

When exposed to solar irradiance, the Micro Solarcopter can potentially stay operational indefinitely. A limitless operating time is possible because the night cycle is not long enough to deplete the charge in the battery completely before the solar panel starts to charge the battery during the day cycle, and the rate of discharge of the battery in sleep mode is considerably slower than the rate of charge during the day cycle.

An Arduino Uno was initially used to test the code for the sleep mode, automatic on and off, LED, and low power alarm, these were subsequently implemented into the system using an ATtiny85 microcontroller. An IRL540N MOSFET capable of handling 28A of continuous current at a transistor-transistor logic (TTL) level of 5 V was used for turning on and off the power to the FPV camera and FC. This TTL level is a requirement of the microcontroller. The MOSFET has been purposely oversized to eliminate the need for a heatsink, which is inherently heavy.

**Figure 6:**
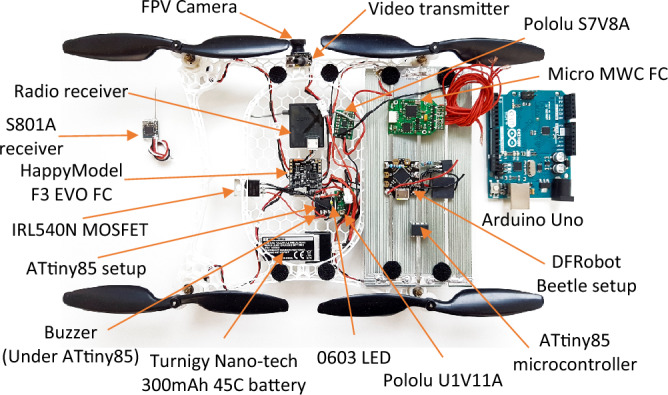
Micro Solarcopter electronic components. The ATtiny85 sits on top of the buzzer, and the LED on top of the Attiny85. This setup also used surface mount resistors compared to the through-hole components used on a DFRobot Beetle setup. The FPV camera is sandwiched onto the video transmitter merging them.

The MOSFET was strategically placed to be exposed to the rotor airflow in flight, providing some level of cooling. MOSFETs lose some power as heat, and it is essential to keep them cool to avoid performance degradation, or damage to surrounding components. To ensure stable power is received, the Micro Solarcopter comprises two switching (step-up/step-down) voltage regulators, one that boosts the battery voltage up to 5 V to operate the ATtiny85 microcontroller, and one to boost the solar panel output to a stable 4 V to charge the lithium polymer battery. A voltage of 4 V was selected to give a safety margin as single-cell lithium-polymer based batteries will become permanently damaged if charged over their maximum voltage of 4.2 V. The electronic components used on the MAV are presented in (Fig. 6).

The voltage regulators used for charging the battery from the solar panel is the Pololu S7V8A. This chip is small and light-weight, with a mass of only $0.0006\mathrm{ kg}$. It utilises the Texas instruments TPS6306x Switch-Mode buck-boost converter and can switch up to 2 A of current with a power conversion efficiency that can reach $93\%$. It has an integrated constant-current constant-voltage (CC-CV) charging method required by the battery. It also has reverse current protection which will ensure that the solar panel does not discharge the battery during periods of no light.

The voltage regulator used for the operation of the ATtiny85 is the Pololu U1V11. This chip can operate at voltages as low as 0.5 V and maintain a stable 5 V output. This chip is useful as it will keep the system operating even if the system experiences a significant voltage drop either from a damaged or overloaded battery.

If the voltage input to the ATtiny85 is not stable, then the Analog to Digital Converter (ADC) on the chip can misread the battery voltage. It can also cause the chip to malfunction, which can be disastrous during flight as it controls the power delivery to the FC and propulsion.

The Micro Solarcopter utilises SunPower C-60 J-Bin monocrystalline cells. This solar cell can achieve an efficiency of 22.5%^23^ and is relatively low cost compared to other solar cells with higher efficiencies. This cell provided the best specific power and power density within the budget constraints. A complete SunPower C60 solar cell is relatively large compared to the Micro Solarcopter. The cell had to be cut to produce a solar panel that is small in size and that met a target power with desired voltage and current properties. Solar cells also require a form of power management to maximise their power output as they follow a characteristic Current–Voltage (IV) curve^35^. Table 2 indicates the property of the 4-cell panel developed for the Micro Solarcopter.

**Table 2:** Micro solarcopter solar panel characteristics.

Parameter	Value
Maximum power	1.65 W
Current at maximum power point	0.71 A
Voltage at maximum power point	2.33 V
Panel efficiency	22%
Single cell (stripe) area (4 cells in panel)	0.015 m $\times$ 0.125 m
Mass (unencapsulated)	0.00352 kg
Cell thickness	0.000165 m

Multiple charge control chipsets were tested to ascertain their operating efficiencies. To ensure uniform testing conditions and facilitate comparison of the chips, the solar panel were simulated using a Chroma 62000H-S series solar array simulator, while the battery was simulated using an Applent AT8612 DC electronic load.

**Figure 7:**
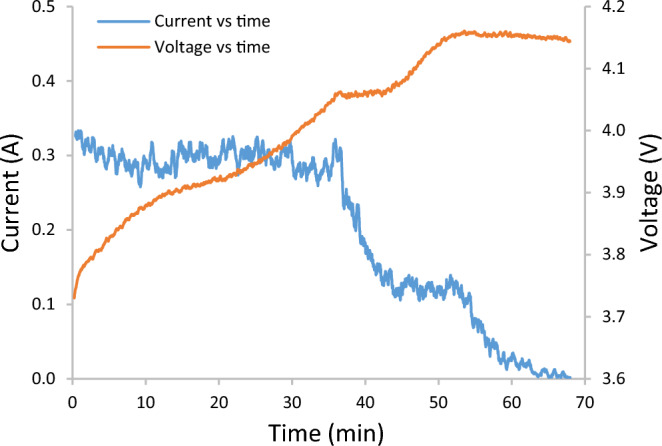
Battery charge time using S7V8A and simulated solar panel under standard test conditions (STC). The charge time for the Turnigy 0.3 Ah battery was found to be approximately 68 min. The DC-DC converter acts as a power supply and charges the battery using a constant current then constant voltage method. The S7V8A chipset was set to just under 4.2 V in order not to overcharge and damage the battery.

It was determined from experiments that a 0.125 m × 0.060 m surface area solar panel tailored to a Pololu S7V8A charge control chipset was the most suitable means of energy generation for the Micro Solarcopter, for its low mass and size, high efficiency and consistent results. While the Pololu S7V8A does not perform maximum power point tracking (MPPT), the solar panel was designed to have its maximum power point as close to the shutdown voltage of the chipset as possible. This design allows the chipset to employ a form of voltage proportional charge control (VPCC) whereby if the voltage of the solar panel falls below a specific voltage value, the current output is reduced to stabilize the voltage. In the case of the Pololu S7V8A, the chipset shuts down when the voltage is too low and turns back on when the voltage recovers, ensuring the solar panel operates close to its maximum power point voltage. The best match was a four-cell setup connected in series. The resulting total power conversion efficiency of this setup is 70%. The charge time of the selected battery was then determined experimentally using the solar array simulator and electronic load, the results can be seen in (Fig. 7).

The frame of the Micro Solarcopter is required to securely hold all components while withstanding mechanical and thermal stresses caused by the propulsion, solar cell, battery, and electronics. The solar cell can become substantially hot if left sitting for long periods in the sun. The motors of the propulsion system also tend to heat up during the flight. The MOSFET and battery can become considerably warm under high power loading conditions such as during periods of full throttle and high-speed flight. Furthermore, the flight controller requires a rigid frame with vibration dampening to minimize noise affecting the sensitive IMU sensors. Finally, the frame should not obstruct the airflow of the propellers. The frame of the aircraft was realised using Autodesk Inventors generative design feature and was made from Polylactic acid (PLA).

Constraints and inputs used included the volume that the frame was to fit in, forces and moments it should withstand and properties of materials to be utilised. This design was then 3D-printed and improved iteratively. The final design consisted of two perimeter shells, with zero top and bottom solid layers. The removal of these layers would improve cooling within the structure relative to a structure with closed top and bottom layers. The increased cooling is due to the increased surface area in contact with the propeller airflow acting similar to radiator fins. The overall mass of the structure is also reduced due to the removal of the top and bottom layers. The mass distribution of the micro Solarcopter is presented in Table 3.

**Table 3:** Mass distribution for MAV components.

Component	Mass [g]	Mass [%]
3D printed (PLA) frame	12.40	17.34
3D printed (PLA) landing gear $\times$ 4	0.92	1.29
Propeller (hobbyking 5030) $\times$ 4	4.97	6.95
Motors (nine eagles) $\times$ 4	19.60	27.41
Gear box (nine eagles 4:1) $\times$ 4	5.52	7.72
Solar panel (encapsulated)	5.50	7.69
Flight control (F3 evo micro)	2.00	2.80
Spektrum satellite reciever	3.30	4.61
Battery	8.00	11.19
FPV Camera (Micro AIO 5.8 GHz)	3.60	5.03
ATtiny 85	0.50	0.70
Polulu U1V11A	0.70	0.98
Polulu S7V8A	0.60	0.84
IRL540N	1.97	2.75
Murata 75 kHz 4db buzzer	0.85	1.19
Mechanical power switch	0.08	0.11
29AWG stranded copper wire	1.00	1.40
Total mass	71.51	

## The vehicle in operation

The Micro Solarcopter can be seen in flight in (Fig. 1). The radio-controlled 0.15 m × 0.15 m × 0.02 m aircraft can travel over 15 m in height and range with a flight endurance of 3.5 min depending on the defined voltage cut-off setting which directly impacts battery cycle life. A higher voltage cut-off decreases flight time but ensures a longer battery cycle life while a lower battery cut-off setting ensures a longer flight time but shorter battery cycle life.

For this setup, it has been set to 3 V which reduces the flight time as the battery recovers to around 3.8 V. This is partly due to voltage sag caused by high current draw during flight.

The FPV camera installed onboard the Micro Solarcopter transmits live video feed to the operator through FPV goggles or a monitor. This capability gives the Micro Solarcopter operator a cockpit view while flying the aircraft. The FPV combination utilized a micro on-board camera and a 25mW 5.8 Ghz video transmitter. A battery recharge time of 68 min was achieved using a solar array simulator with the solar panel specifications under standard test conditions of irradiance and 25 °C.

The design of the Micro Solarcopter involved a balancing act between system efficiency with regards to low disk loading propellers, and stability and control of the aircraft, as it weighs only 0.071 kg. The Micro Solarcopter can generate a maximum axial velocity of 5.2 m/s ± 5% from its propellers with a thrust-to-weight ratio of 1.93:1.

The terminal velocity of the Micro Solarcopter was found to be 6.23 m/s. Therefore, the Micro Solarcopter has a terminal momentum of only 0.442 kgm/s and terminal kinetic energy of 1.39 Nm. The low mass and momentum of this system means it is well below the injury threshold (category 2) of the FAA requirements. The low momentum value is due to the increasing ratio between air drag and mass, as the system is scaled down in size^21^, making the aircraft less destructive if it were to fail during a flight and fall to earth. The Micro Solarcopter has a 300 mAh battery and a microcontroller that wakes up every 8 s resulting in 450 wakeups per hour. These components enable the aircraft to hibernate for approximately 38 days without its solar panel exposed to sunlight.

## Discussion

It has been demonstrated that a solar-rechargeable multi-rotor MAV is a feasible platform for applications ranging from surveillance to atmospheric monitoring through the realisation of a working prototype. The applications are, however, limited as a result of its physical size.

These limitations are due to the adverse effects of scaling down such as a low Reynolds number leading to a lower lift to drag ratio, requiring a greater induced power to attain flight, and the limitation in present solar cell and propulsion technologies. The aircraft flew and maintained a stable hover with good control for an average flight time of 3.5 min. This flight time is short compared to fixed-wing MAVs but close to multi-rotor MAV endurance figures of around 5 minutes. The advantage, however, is that the Micro Solarcopter can land, hibernate and independently recharge its battery without having to return to a home base.

Solar panel surface area and propulsion performance were found to be the two main limiting factors towards producing a purely solar-powered multi-rotor MAV without energy storage. The required panel area would exceed MAV dimensions of 0.15 m × 0.15 m to provide enough power necessary to generate enough lift for hover.

A purely solar-powered rotary wing MAV is not feasible with current technology and would not be practical for real-world applications due to the large solar panel surface area to total system mass ratio that would be required, coupled with the low generated dynamic pressure of MAVs, making the concept highly susceptible to wind gusts. As the thrust output of MAVs is limited by their size, they, therefore, cannot fly in high wind speeds, on the other hand, however, they are more robust and crash-resistant than their larger counterparts.

These characteristics are in-line with the flight test results of the Micro Solarcopter, as the aircraft did have difficulty flying in high wind speeds, and at times would fall into a vortex-ring state. This condition applies mainly to rotorcraft where the aircraft falls into its downwash leading to a loss of lift, resulting in a loss of stability and control of the aircraft. The cause of the condition with regards to the Micro Solarcopter could be either down to pilot flying style or the dynamic pressure of the wind pushing the aircraft into its downwash.

The maximum axial wind velocity generated by the Micro Solarcopter propellers was 5.2 m/s ± 5%. The Mach number of the Micro Solarcopter was found to be 0.0151 (± 5%), generating a maximum dynamic pressure of 16.3 N/m^2^ (± 10%), while the disk loading of the Micro Solarcopter at hover is 13.84 N/m^2^ (± 4.96%). This result shows that the wind can substantially affect the stability and control of the Micro Solarcopter, as the aircraft may not be able to escape its effects due to the low dynamic pressure it can generate relative to that which can be generated by the wind on the aircraft. Larger aircraft can generate greater dynamic pressure, which tends to increase with size. While this can be an adverse effect for MAVs, delicate flying insects such as butterflies or moths, which have extremely low wing loadings and generate very low dynamic pressures, are still able to survive and operate in nature even within the effects of wind.

Another limitation of a purely solar-powered MAV design would be the lack of a stable power source as the generated power from the solar panel is dependent on several continuously changing factors such as panel inclination angle, solar irradiance, temperature, and shadows cast by clouds or objects in the environment.

The recharging challenges addressed by the Micro Solarcopter may alternatively be overcome by landing on a moving unmanned ground vehicle where the landing surface is itself the solar panel instead, and the system can, therefore, recharge rapidly and take off again.

## Conclusion and future work

In this article, the effects of solar-powered multi-rotor miniaturisation on performance characteristics such as the flight and charge times, were determined to assess the feasibility of the system for real-world applications. Engineering challenges such as the design of a lightweight rigid frame to house all components have been overcome.

Low-power, high-efficiency propulsion components as well as electronics for flight and charge control that fit within the size and weight restrictions for an MAV were investigated. The selection, sizing, and manufacture of small tailored solar panels, to meet system power requirements while minimising the impact on flight performance due to factors such as increased system mass and size have also been demonstrated. The sizing and selection of an appropriate power storage solution has also been determined.

The final miniaturised working prototype has been equipped with intelligent features such as going into a hibernation mode when its battery is depleted, and automatically waking up when the battery becomes fully charged by solar power. These are the first steps towards a fully autonomous solar-powered multi-rotor MAV. A first person view (FPV) camera is included onboard the aircraft to demonstrate that additional sensors can be attached depending on the intended application. A future vision for this system is to create a swarm workforce^17^ of completely autonomous MAV robots^18^ that can be put into service and only return from field duties during maintenance periods rather than also to recharge.

The Micro Solarcopter provides a starting point for the future development of renewable-energy powered flying multi-rotor MAVs. There are benefits to multi-rotors over fixed-wing aircraft such as the ability to hover, low-speed and low-level flight which makes them better suited for particular applications. The addition of an appropriately sized solar panel does increase the energy autonomy of these systems even at micro scales, however, larger systems will benefit more due to the scaling effects described earlier.

Automatic control would be a logical next step towards conducting missions autonomously. Based on the performance of this MAV, the most suitable applications could include routine low-level flight surveillance, tracking or inspection, with either real-time data relay or storage for offline analysis. Beyond autonomous control, further development could be implementing a swarm of these aerial robots to collaborate on a mission. The mission duration could last substantially longer or be continuous, providing a real-time continuous flow of data to its users without requiring the swarm to return to base due to its energy autonomy (Video S1).

### Supplementary Information

Supplementary Video 1.

Supplementary Information 1.

## Data Availability

The data that support the findings of this paper are available from the corresponding author upon reasonable request.

## References

[1] Schoeberl, E. From sunrise to solar-impulse 34 years of solar powered flight. (2008).

[2] Siegwart, R., Mattio, A., Bouabdallah, S. & Gros, S. Modelling and Control of the UAV Sky-Sailor. *Sky-Sailor.Ethz.Ch* 1–88 (2006).

[3] Liew, C. F., DeLatte, D., Takeishi, N. & Yairi, T. Recent developments in aerial robotics: A survey and prototypes overview. 1–14 (2017).

[4] Sunier, J. *et al.* Innovative PV modules for Atlantic ocean crossing AtlantikSolar: First solar-powered and fully autonomous crossing of the Atlantic Ocean High end materials. 5700 (2015).

[5] Kumar, A. Aquila (the Solar Powered Drone ). *International Journal of Industrial Electronics and Electrical Engineering* 36–43 (2016).

[6] Abidali, A. & Shaheed, M. H. *Design and construction of a solar powered remote controlled helicopter (Project report)*. (2012).

[7] Shaheed MH (2015). Flying by the Sun only: The solarcopter prototype. Aerosp. Sci. Technol..

[8] Jr, H. C. L. *et al.* Development of a remote-controlled quadrotor with solar recharging and emergency landing capabilities. (2016).

[9] Pramod H (2017). Design and development of solar powered quadcopter using 3D printing technology. Int. J. Eng. Res. Appl..

[10] Teo, B. S., Henz, M. & Danner, A. J. Solar powered rotorcraft: A multidisciplinary engineering challenge for undergraduate students. In *14th Conference on Education and Training in Optics and Photonics: ETOP 2017* **10452**, 49 (2017).

[11] Goh CS, Kuan JR, Yeo JH, Teo BS, Danner A (2019). A fully solar-powered quadcopter able to achieve controlled flight out of the ground effect. Prog. Photovolt. Res. Appl..

[12] Veismann M, Dougherty C, Rabinovitch J, Quon A, Gharib M (2021). Low-density multi-fan wind tunnel design and testing for the Ingenuity Mars Helicopter. Exp. Fluids.

[13] Elkunchwar, N., Chandrasekaran, S., Iyer, V. & Fuller, S. B. Toward battery-free flight: Duty cycled recharging of small drones. in *IEEE International Conference on Intelligent Robots and Systems* 5234–5241 (Institute of Electrical and Electronics Engineers Inc., 2021). 10.1109/IROS51168.2021.9636087.

[14] Kroo, I., & Kunz, P. Development of the mesicopter: A miniature autonomous rotorcraft. *Vertical Lift Aircraft Design Conference (A00–26651 06–05)* 1–9 (2000).

[15] Grasmeyer JM, Keennon MT (2001). Development of the black widow micro air vehicle. AIAA.

[16] Kushleyev, A., Mellinger, D. & Kumar, V. Towards a swarm of agile micro quadrotors (2012).

[17] Augugliaro F (2014). The flight assembled architecture installation: Cooperative contruction with flying machines. IEEE Control Syst..

[18] Palossi, D. *et al.* Ultra low power deep-learning-powered autonomous nano drones. 1–12 (2018).

[19] Noth A, Siegwart R, Engel W (2007). Design of solar powered airplanes for continuous flight. Environ. Res..

[20] Tsoutsos T, Frantzeskaki N, Gekas V (2005). Environmental impacts from the solar energy technologies. Energy Policy.

[21] Estier, C., & Siegwart. Fascination of down scaling—Alice the sugar cube robot. *J. Micromechatronics* **1**, 177–189 (2001).

[22] Zulu A, John S (2014). A review of control algorithms for autonomous quadrotors. Open J. Appl. Sci..

[23] SunPower Corp. C60 Solar Cell Mono Crystalline Silicon. *Sunpower Corporation* 3–4 (2010).

[24] Shockley W, Queisser HJ (1961). Detailed balance limit of efficiency of p-n junction solar cells. J. Appl. Phys..

[25] Altas IH, Sharaf AM (2014). Solar energy and PV systems. Int. J. Photoenergy.

[26] Duffie, J. A., & Beckman, W. A. *Solar engineering of thermal processes solar engineering*. 10.1002/9781118671603.fmatter (2013).

[27] Bouabdallah S (2007). Design and control of quadrotors with application to autonomous flying. École Polytechnique Fédérale De Lausanne, À La Faculté Des Sciences Et Techniques De L’Ingénieur.

[28] Noth, A. Design of solar powered airplanes for continuous flight (PhD Thesis - Noth 2008). (1980).

[29] Johnson, W. Helicopter theory. *New York* 1–64. 10.1163/_q3_SIM_00374 (1980).

[30] Ramasamy M, Leishman JG, Lee TE (2007). Flowfield of a rotating-wing micro air vehicle. J. Aircr..

[31] Scherz, P. & Monk, S. *Practical Electronics for Inventors*. (Cenveo Publisher Services, 2016).

[32] Bresciani T (2008). Modelling, identification and control of a quadrotor helicopter. English.

[33] Survey, E. U. M. Signal Processing. in *Encyclopedia of Electronic Components* (ed. Jepson, B.) vol. 2 316 (Maker media, 2009).

[34] Honsberg, C. & Bowden, S. Effect of Temperature | PVEducation. *pveducation.org* 4–6 https://www.pveducation.org/pvcdrom/solar-cell-operation/effect-of-temperature.

[35] Sze SM, Kotz S, Nash FR, Bossard PR (1989). IEEE transactions on electron devices. IEEE Trans. Electron Dev..

