# Supplementary material for: Development of a solar powered multirotor micro aerial vehicle
Source: Sci Rep. 2024 Mar 8;14:5771. doi: 10.1038/s41598-024-54079-9 (PMC10923847; doi:10.1038/s41598-024-54079-9)
Supplement: Supplementary file 2 — Supplementary Information 1. [file 41598_2024_54079_MOESM2_ESM.docx]

Supplementary Materials for

Development of a solar powered multirotor micro aerial vehicle

Aly Abidali, ^1^ Stephen A. Agha, ^1^ Ante Munjiza, ^1^ Mohammad H. Shaheed^1^,*

Correspondence to: m.h.shaheed@qmul.ac.uk

**This PDF file includes:**

Figs. S1 to S4

Tables S1 to S2

Caption for Movie S1

**Other Supplementary Materials for this manuscript include the following:**

Movie S1


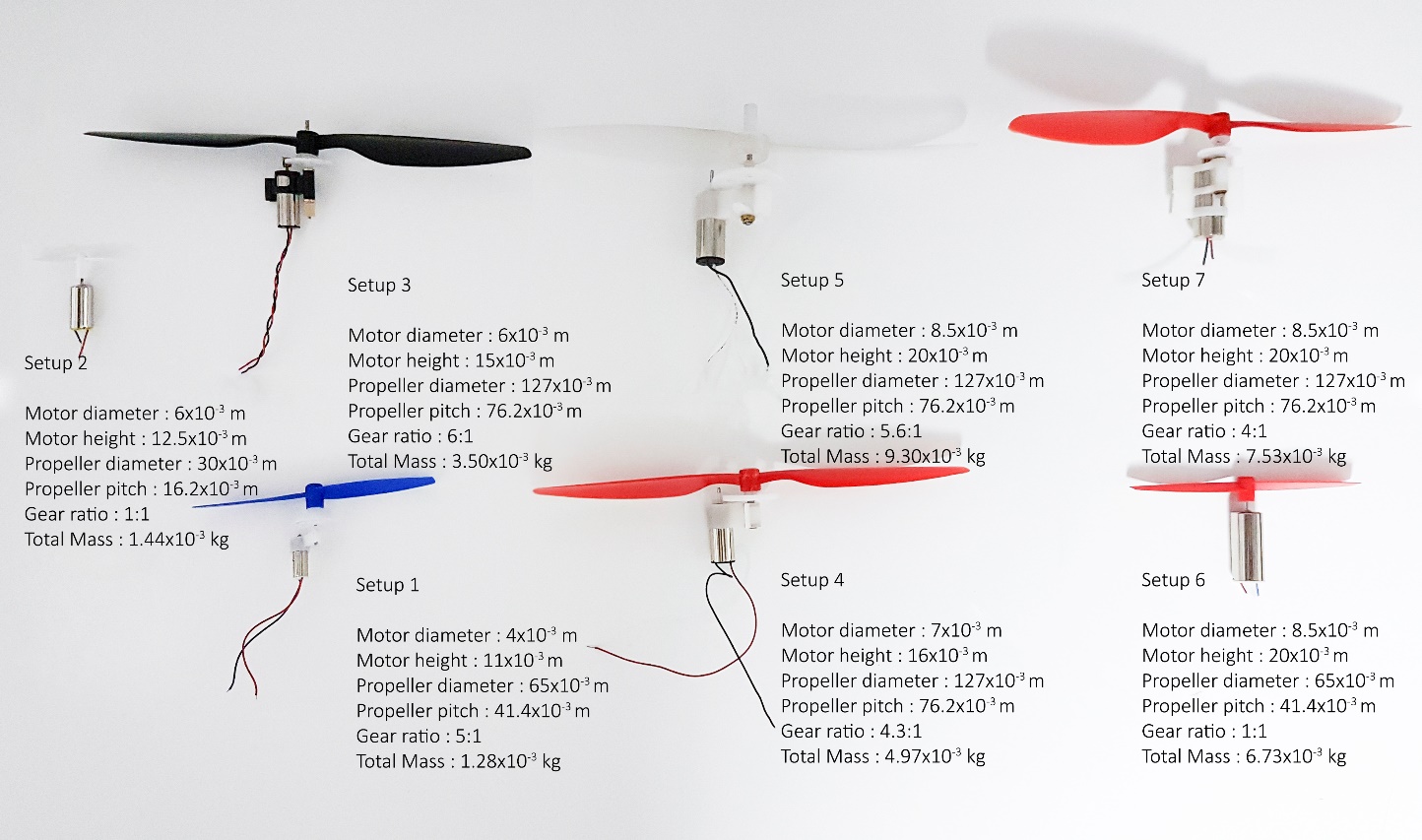


Fig. S1. Propulsion system combinations.

The motor and propeller combinations selected for testing were adapted from aircraft off similar scales to that of the design criteria. The dimensions in the illustration show the diameter of the motor by its length.

The 6$\times$10^-3^m diameter by 12.5$\times$10^-3^m height motor with the 30$\times$10^-3^m diameter propeller was disassembled from the M:Tech Micro quadcopter.


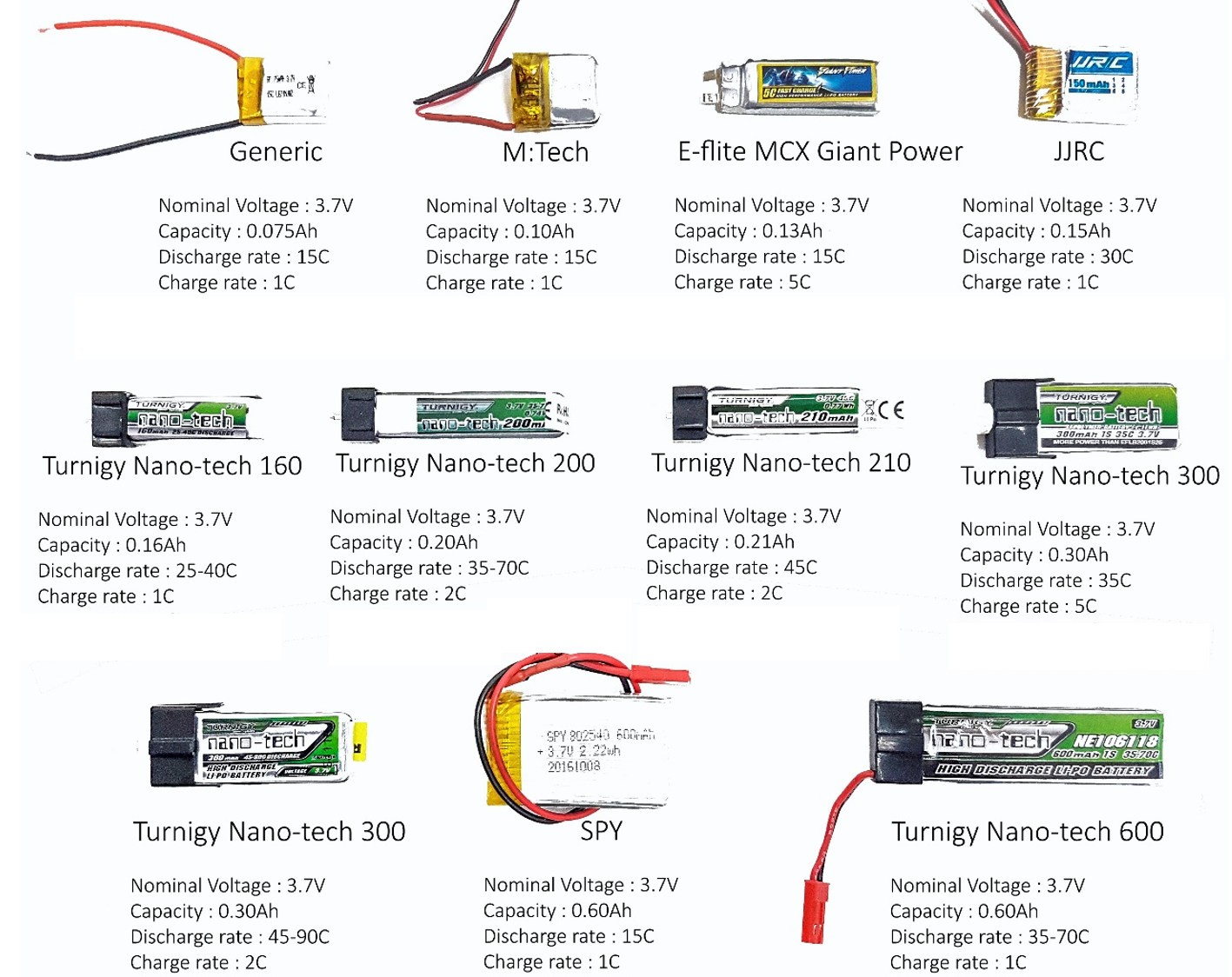


Fig. S2. Energy storage candidates.

The batteries tested were all advertised as lithium-polymer chemistries. Most of the batteries underperformed in loading experiments with regards to their discharge rate. This finding was observed with a substantial voltage drop when the system was under load. The two most suitable candidates found were the Turnigy Nano-tech 0.3Ah, 45-90C and 0.6Ah, 35 – 70C, for load currents ranging from 5A and above.

| **Battery Name** | **C rating** | **Battery**  **Capacity**  **(x10^-3^Ah)** | **Battery dimensions**  ***h* x *w* x *d***  **(x10^-3^m)** | **Battery mass**  **(x10^-3^kg)** | **Battery volume**  **(x10^-9^m^3^)** | **Specific power (W/kg)** | **Specific energy (Wh/kg)** | **Power density**  **(x10^6^W/m^3^)** | **Energy**  **density**  **(x10^5^Wh/m^3^)** |
| --- | --- | --- | --- | --- | --- | --- | --- | --- | --- |
| **Generic** | 15 | 75 | 14.65x18.96x6.00 | 2.72 | 1667 | 1530 | 102 | 2.50 | 1.66 |
| **M:Tech** | 15 | 100 | 15.84x18.47x7.76 | 3.55 | 2270 | 1563 | 104 | 2.44 | 1.63 |
| **E-flite MCX** | 15 | 130 | 11.52x35.89x5.58 | 3.77 | 2307 | 1914 | 128 | 3.13 | 2.08 |
| **JJRC** | 30 | 150 | 17.00x25.41x8.08 | 5.57 | 3490 | 2989 | 100 | 4.77 | 1.59 |
| **Turnigy 160** | 25 | 160 | 10.36x36.34x6.56 | 3.55 | 2470 | 4169 | 167 | 5.99 | 2.39 |
| **Turnigy 200** | 35 | 200 | 11.20x50.98x5.88 | 5.90 | 3357 | 4390 | 125 | 7.71 | 2.20 |
| **Turnigy 210** | 45 | 210 | 11.45x43.81x6.31 | 5.85 | 3165 | 5977 | 133 | 11.05 | 2.45 |
| **Turnigy 300** | 35 | 300 | 16.48x43.22x6.10 | 8.07 | 4345 | 4814 | 138 | 8.94 | 2.55 |
| **Turnigy 300** | 45 | 300 | 16.06x43.45x6.17 | 8.00 | 4305 | 6244 | 139 | 11.60 | 2.57 |
| **SPY** | 15 | 600 | 24.37x40.46x8.23 | 17.26 | 8115 | 1929 | 129 | 4.10 | 2.73 |
| **Turnigy 600** | 35 | 600 | 17.25x65.10x7.17 | 15.65 | 8052 | 4965 | 142 | 9.65 | 2.75 |

**Table S1:** Battery parameters calculated from advertised ratings

The battery parameters of selected batteries. The table has been colour-coded based on the relative magnitudes for each of the parameters between batteries, with green showing the most desirable, red the least desirable and amber in between. While the Turnigy 160 appeared to be a good candidate on paper, discharge tests of these batteries (such as shown in figure 4 within the paper) indicated that actual C ratings are lower than advertised as the battery voltage drop was dangerously high under these load conditions. The voltage sag can be damaging for the battery and capable of shutting down electronic components, and producing less thrust from the propulsion components. Note that the battery dimensions show the height $h$, the width $w$ and the depth $d$.


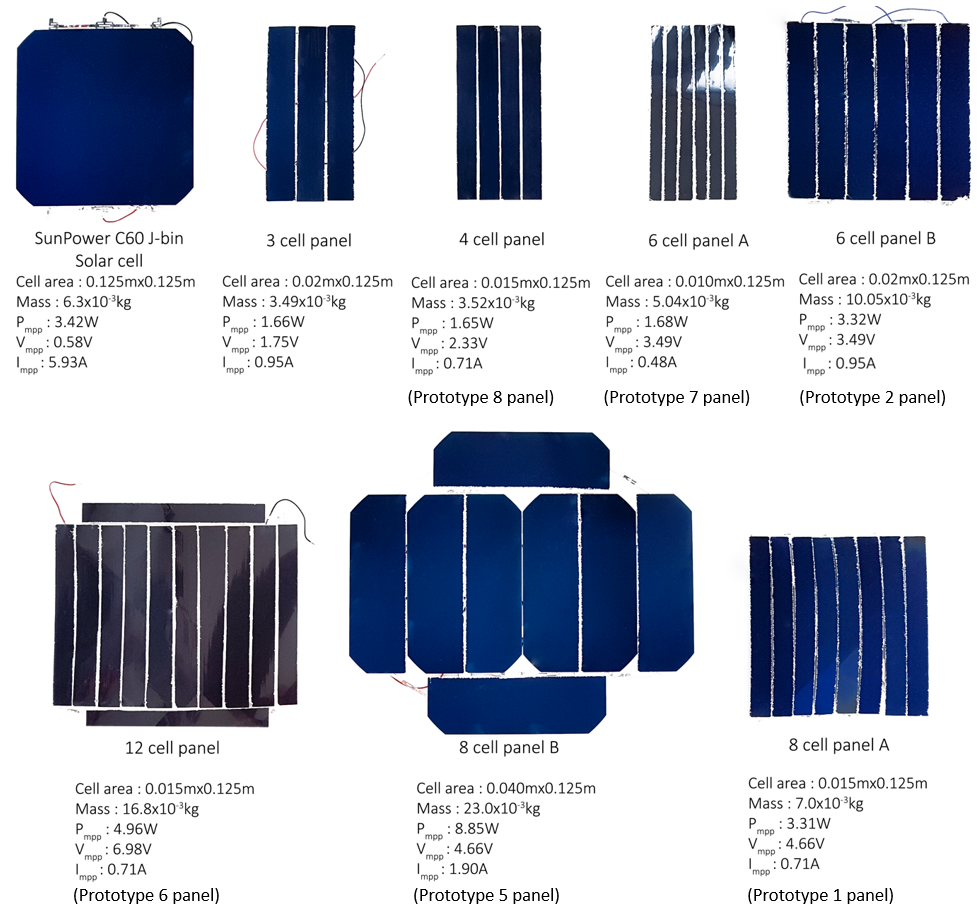


Fig. S3. Manufactured solar panels used for testing.

On the top left corner, a single SunPower C60 J-bin solar cell can be seen in its original form. This cell had to be cut vertically from one connection side to the other to produce solar panels of varying operating voltages and currents as shown. Note that some panels are encapsulated while others are not, affecting their total mass. The mass of the cell tends to increase more than expected as the number of connections and the overall size of the panel increases. This is due to the connections requiring additional tabbing wire and solder increasing the overall mass.


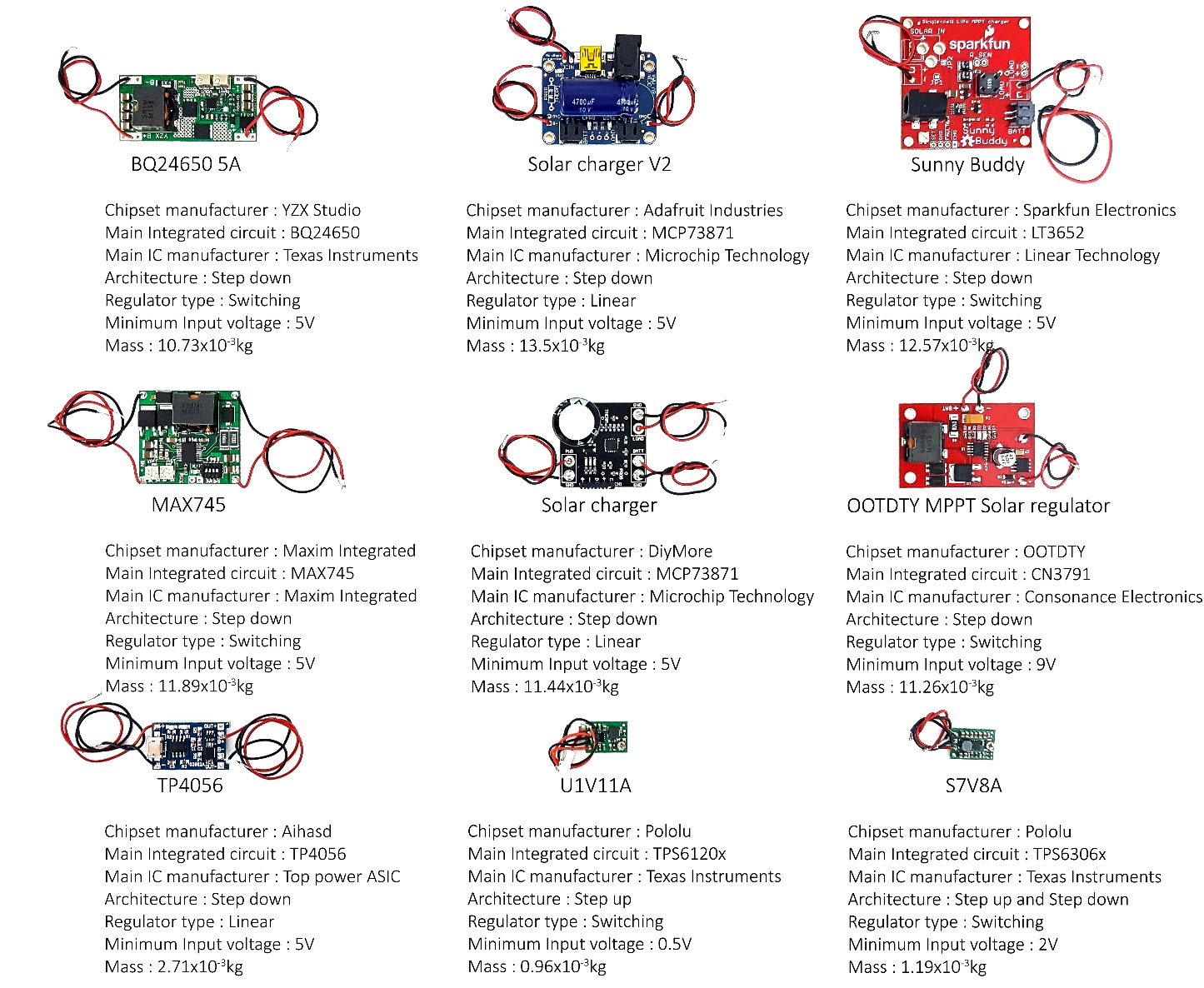


Fig. S4. Charge control chipsets.

These chipsets were tested for maximum power output with minimal impact on flight time. They all have different operating voltages and charge control methods. The BQ24650, Sunny Buddy and OOTDTY employ true Maximum power point tracking while the Solar charger and Solar charger V2 provide voltage proportional charge control. For the MAX745, TP4056, U1V11A and S7V8A a solar panel was tailored for maximum power output by either limiting current output or matching the minimum operating voltage of the chipsets with the maximum power point voltage of the solar panel.

| **Propulsion setup** | **Motor name** | **Gearbox name** | **Propeller name** | **Motor mass**  **(x10^-3^kg)** | **Gearbox mass**  **(x10^-3^kg)** | **Propeller mass (x10^-3^kg)** | **Total mass**  **(x10^-3^kg)** | **Maximum thrust**  **(N)** | **Average *T_W_***  **(N/W)** |
| --- | --- | --- | --- | --- | --- | --- | --- | --- | --- |
| **Setup 1** | Plantraco GB05 | Plantraco GB05 | Parrot RS | 0.649 | 0.225 | 0.401 | 1.275 | 0.061 | 0.053 |
| **Setup 2** | Mtech Micro | N/A | Mtech Micro drone | 1.352 | - | 0.091 | 1.443 | 0.112 | 0.027 |
| **Setup 3** | Parkzone  Vapor | Parkzone Vapor | Turnigy Micro-quad | 1.699 | 0.538 | 1.258 | 3.495 | 0.174 | 0.090 |
| **Setup 4** | Aero Electronics Operations GPS7 | Aero Electronics Operations GPS7 | Turnigy Micro-quad | 2.695 | 1.105 | 1.166 | 4.966 | 0.328 | 0.044 |
| **Setup 5** | Aero Electronics Operations GPS8 | Aero Electronics Operations GPS8 | Diatone | 5.091 | 2.125 | 2.082 | 9.298 | 0.324 | 0.014 |
| **Setup 6** | HobbyKing Mini Quad | N/A | Parrot RS | 5.475 | 0.000 | 0.401 | 5.876 | 0.333 | 0.028 |
| **Setup 7** | Nine Eagles  P-51 micro | Nine Eagles  P-51 micro | Turnigy Micro-quad | 5.143 | 1.124 | 1.258 | 7.525 | 0.372 | 0.042 |

Table S2. Propulsion setups parameters and performance results.

Table 2 shows the parameters of the propulsion system combinations. Some setups have a gearbox while others are direct drive. The average thrust per watt (Average $T_{w}$) is the average of all the positive thrust per watt ($T_{w}$) values across the entire power band excluding negative values when factoring in propulsion system mass. (Thrust is considered positive once the thrust generated by the propulsion system is sufficient to overcome its own weight). Propulsion systems were then chosen based on the Average thrust per watt and the maximum thrust. The data was collected using a load cell calibrated using a precise 20-gram mass.

Movie S1.

This movie shows the results of the Micro Solarcopter flight tests. The stability and control of the aircraft can be perceived including first-person view footage, flight endurance, and the vortex-ring state condition experienced by the aircraft.

Due to the length of footage, the video quality had to be substantially reduced to meet the submission size requirements. A link has been provided below to view a high definition version of the movie for the reader’s clarity:

<https://1drv.ms/v/s!Apowx5SblGoegbBbbUdXA05UgRMgHg>

Only author (s) were involved in performing the experiments.
